# Genomic stability and differential expression of type V cif genes in *Wolbachia* with variable cytoplasmic incompatibility phenotypes in spider mites

**DOI:** 10.1093/ismeco/ycag263

**Published:** 2026-09-11

**Authors:** Xiao-Li Bing, Shuo Gao, Jia-Mei Li, Lei Chen, Huan Wang, Yu Liu, Huan-Huan Liu, Xiao-Yue Hong

**Affiliations:** Sanya Institute of Nanjing Agricultural University, State Key Laboratory of Agricultural and Forestry Biosecurity, Key Laboratory of Integrated Management of Crop Disease and Pests (Ministry of Education), College of Plant Protection, Nanjing Agricultural University, Nanjing, Jiangsu 211800, China; Sanya Institute of Nanjing Agricultural University, State Key Laboratory of Agricultural and Forestry Biosecurity, Key Laboratory of Integrated Management of Crop Disease and Pests (Ministry of Education), College of Plant Protection, Nanjing Agricultural University, Nanjing, Jiangsu 211800, China; Sanya Institute of Nanjing Agricultural University, State Key Laboratory of Agricultural and Forestry Biosecurity, Key Laboratory of Integrated Management of Crop Disease and Pests (Ministry of Education), College of Plant Protection, Nanjing Agricultural University, Nanjing, Jiangsu 211800, China; Sanya Institute of Nanjing Agricultural University, State Key Laboratory of Agricultural and Forestry Biosecurity, Key Laboratory of Integrated Management of Crop Disease and Pests (Ministry of Education), College of Plant Protection, Nanjing Agricultural University, Nanjing, Jiangsu 211800, China; Sanya Institute of Nanjing Agricultural University, State Key Laboratory of Agricultural and Forestry Biosecurity, Key Laboratory of Integrated Management of Crop Disease and Pests (Ministry of Education), College of Plant Protection, Nanjing Agricultural University, Nanjing, Jiangsu 211800, China; Sanya Institute of Nanjing Agricultural University, State Key Laboratory of Agricultural and Forestry Biosecurity, Key Laboratory of Integrated Management of Crop Disease and Pests (Ministry of Education), College of Plant Protection, Nanjing Agricultural University, Nanjing, Jiangsu 211800, China; Sanya Institute of Nanjing Agricultural University, State Key Laboratory of Agricultural and Forestry Biosecurity, Key Laboratory of Integrated Management of Crop Disease and Pests (Ministry of Education), College of Plant Protection, Nanjing Agricultural University, Nanjing, Jiangsu 211800, China; Sanya Institute of Nanjing Agricultural University, State Key Laboratory of Agricultural and Forestry Biosecurity, Key Laboratory of Integrated Management of Crop Disease and Pests (Ministry of Education), College of Plant Protection, Nanjing Agricultural University, Nanjing, Jiangsu 211800, China

**Keywords:** *Wolbachia*, *Tetranychus urticae*, cytoplasmic incompatibility, spider mite, CI factors

## Abstract

The two-spotted spider mite, *Tetranychus urticae*, is a globally significant agricultural pest frequently infected by the endosymbiont *Wolbachia*. *Wolbachia* induces cytoplasmic incompatibility (CI) with varying intensity across geographic populations. Despite the importance of CI, its molecular mechanisms in *T. urticae* remain poorly understood. Here, we demonstrate that CI differences are driven by both *Wolbachia* (*w*Turt) strain variations and host nuclear background. The *w*Turt genome harbors two pairs of *cif* genes (*cifA1-B1* and *cifA2-B2*), both phylogenetically classified as Type V. Genomic analysis revealed no SNP differences in either pair of *cif* genes in *w*Turt infecting different geographic populations of spider mites. While *cifA1-B1* is barely expressed, *cifA2-B2* exhibits high expression levels that vary significantly across multiple *T. urticae* populations, with expression levels positively correlated with *w*Turt density. Yeast two-hybrid experiments revealed physical interactions between CifA2 and the PD-(D/E)XK nuclease domain of CifB2, suggesting that this pair may contribute to CI modulation. Codon optimization revealed that the CifB2 nuclease domain is toxic in yeast, but this toxicity was not rescued by CifA2. Transgenic Drosophila assays likewise failed to reproduce CI-related phenotypes. Altogether, our findings advance the understanding of *Wolbachia*-host reproductive manipulation and provide insights for symbiont-based biocontrol strategies for agricultural mite pests.

## Introduction

Symbiotic bacteria are widely present in arthropods. They influence host physiology and reproductive performance through various mechanisms, with their regulation of reproduction being particularly significant. This is most evident in phenotypic traits such as parthenogenesis, male-killing, feminization of genetic males, and cytoplasmic incompatibility (CI). Among these reproductive strategies, CI has become a central topic of long-standing scientific interest due to its potential as a biological control strategy, capable of suppressing pest populations [1]. CI indicates that when a symbiont-infected male mates with an uninfected female or a female infected with a different strain, fertilized embryos often experience developmental abnormalities, leading to reduced or even zero hatching rates, or male-biased sex ratio in haplodiploid species [2]. However, when a symbiont-infected male mates with a female infected with the same strain, the symbiont in the female’s egg cells can “rescue” the embryonic death, allowing normal development to resume [3].

Numerous symbiotic bacteria known to induce CI have been identified, including *Wolbachia* (first reported in 1971) [4], *Cardinium* (2003) [5], *Mesenetia* (formerly known as *Mesenet*) (2017) [6], *Rickettsiella* (2020) [7], *Spiroplasma* (2022) [8], *Rickettsia* (2024) [9], and *Lariskella* (2025) [10]. Current research on CI molecular mechanisms mainly focuses on *Wolbachia*-induced classic CI phenotypes, due to their clear effects and well-established research systems. The molecular mechanisms underlying *Wolbachia*-induced CI have attracted widespread attention, including multi-omics comparative analyses and detailed cytogenetic studies [11–14]. Recent studies have revealed that two adjacent genes in the *Wolbachia* or *Wolbachia*-associated prophage genome, cifA (CI factor A, also referred to as cidA or cinA in some literature) and cifB (CI factor B, also referred to as cidB or cinB), are the key effector factors mediating CI [15–17]. Functional validation of CifA and CifB as core CI factors has been primarily demonstrated through molecular biology experiments. Yeast toxicity assays show that yeast *Saccharomyces cerevisiae* expressing CifB alone exhibits significant survival defects (similar to a toxic effect). However, when both CifA and CifB are co-expressed, yeast toxicity is reversed (i.e. the “rescue” phenomenon) [15, 17]. *In vitro* protein interaction assays further confirmed that CifA and CifB can directly bind to each other [15, 18], suggesting that their interaction regulates cell survival, providing direct evidence for the distinct roles of CifA and CifB as CI effector factors. Using transgenic *Drosophila* systems, CifA and CifB were expressed in *Wolbachia*-uninfected (WU) flies. When these transgenic males mated with uninfected females, the embryos exhibited chromosome bridges and died early in development [16]. When WU female flies express CifA, they can reverse the embryo death induced by CifB (i.e. the “rescue” effect) [19]. These experimental approaches provide core evidence for the crucial role of CifA and CifB in mediating CI, and have become important technical support for understanding the molecular mechanisms of CI.

At the mechanistic level, the core of CI induction by Cif proteins depends on the functional domains they encode, including the DUB (deubiquitinase) domain and the PD-(D/E)XK nuclease domain of CifB [20, 21]. The activity of these domains directly regulates gamete modification and the embryo rescue process, forming the key molecular foundation for the CI phenotype. Notably, current research has revealed significant diversity in Cif proteins, which may sort into as many as 10 distinct types or clades [22–24]. The function of Cifs has been verified in the *Wolbachia* strains of the type I, III, and IV groups [3], while the newly discovered groups, though carrying Cif-related domains, still require further experimental confirmation of their functions.

It is well-known that *Wolbachia* widely infects the two-spotted spider mite (*Tetranychus urticae*) [25]. *Tetranychus urticae* is a globally distributed major agricultural pest that poses persistent threats to agriculture due to its wide host spectrum and strong resistance to multiple pesticides [26]. *Wolbachia* exerts multidimensional regulatory effects on the mite—such as inducing CI to alter host reproductive patterns and influencing key physiological processes like insecticide sensitivity and salivary protein secretion [27, 28]. While our earlier transcriptomic analysis identified gene expression changes associated with CI in *T. urticae* embryos (e.g. significant differential expression of genes related to tissue morphogenesis, DNA damage, and mRNA surveillance) [29], the molecular mechanisms underlying *Wolbachia*-induced CI in this pest remain largely unclear. Notably, the presence and functional characteristics of *Wolbachia*’s key CI-inducing effectors (CifA and CifB) in *T. urticae* warrant further investigation.

In this study, we investigated *Wolbachia*-induced CI phenotypes and their variability within natural haplodiploid *T. urticae* populations. Genomic sequencing of the mite’s *Wolbachia* strain (*w*Turt) revealed the presence of *cifA* and *cifB* genes, which phylogenetic analysis classified as type V. To explore the functional roles of these genes, we employed yeast two-hybrid assays to detect CifA-CifB interactions and conducted yeast toxicity assays as well as transgenic *Drosophila* models. This integrated approach provides new insights into the molecular mechanisms underlying *Wolbachia*-induced CI, thereby advancing our understanding of this widespread reproductive manipulation in arthropods.

## Materials and methods

### Spider mite husbandry

A total of five wild *Tetranychus urticae* spider mite populations were collected from 3 provinces in China, in the summer of 2021 (Table S1). All spider mites were reared on soybean leaves (*Phaseolus vulgaris*) on a water-saturated sponge mat in a Petri dish in the lab after collection [30]. The lab is set with 25 ± 1°C, 16 hour-light photoperiod and 60 ± 10% relative humidity.

### Diagnostic PCR assays of symbiont infection

The infection status of *Wolbachia* was checked by PCR amplification of the *Wolbachia* surface protein (*wsp*) gene with primers 81F and 691R (Table S2) [31]. Total DNA was extracted from individual spider mites using the sodium chloride-Tris-EDTA (STE) buffer containing 1 mg/ml proteinase K as described before [32]. The PCR amplifications were conducted with the 2× Rapid Taq Master Mix (Vazyme Biotech, Nanjing, Jiangsu, China) using an Eppendorf Mastercycler Nexus Thermal Cycler (Eppendorf, Hamburg, Germany).

### Preparation of *Wolbachia*-infected and -uninfected lines

The *T. urticae* Inner Mongolia (NM) populations that were 100% infected or 100% uninfected were previously established in the lab [33]. For new populations, we identified *Wolbachia*-infected (WI) lines using the method from Hong *et al.* [34]. A teleiochrysalis female was collected to reproduce unfertilized eggs. After the eggs developed into males, these males were backcrossed with the mother for 2 days. The mated female was then transferred to a new leaf to lay eggs. We sampled 30–40 offspring and determined the *Wolbachia* infection rate by amplifying the *wsp* gene. A 100% infection rate confirmed successful line establishment.

### Effect of *Wolbachia* infection on mite fecundity, hatch rate, sex ratio, and CI phenotype

A newly emerged virgin female was paired with two males for two days. After mating, the males were removed, and the female laid eggs for three more days. The number of eggs, hatched eggs, and adult male and female offspring were recorded to calculate fecundity, embryonic mortality, and sex ratio. Virgin males (1-day-old) were used to avoid aging or repeated mating effects [35]. More details of gene prediction and annotation are provided in the “Supplementary Materials and Methods”.

For CI phenotypes, two crosses were performed: (i) female WU × male WI (CI cross) and (ii) female WI × male WI (control cross). The WU females in the CI cross were from the NM population. CI intensity was calculated using the following:


$$ \mathrm{CI}\ \mathrm{intensity}=\left(1-\frac{F}{F_{\mathrm{c}}}\right)\times 100 $$

where *F* is the mean number of female adults in the CI cross, and *F*c is the mean in the control cross [36]. Statistical analysis was performed using the Wilcoxon test (CI vs. Control) and the Kruskal–Wallis test with Bonferroni correction for different populations. All analyses were done using R 4.3 [37].

### Preparation of *Wolbachia*-infected lines of the same genetic background

To generate WI populations with the same genetic background but different *Wolbachia* strains, we crossed WI wild females with WU-NM males for eight generations to homogenize the genetic background before bioassays [35]. For example, a wild WI-NJ female (P) was mated with WU-NM males to produce F1 females, which were then mated with new WU-NM males to produce F2, continuing for seven generations. After this, we obtained a line with the NM nuclear genome and the *Wolbachia* NJ strain. Genetic hybridization experiments followed the same procedure. The term “nuclear^Wolbachia^” refers to the mite’s nuclear background and *Wolbachia* strain, e.g. NM^JN^ for NM nuclear background with the JN *Wolbachia* strain. For details, refer to Fig. S1.

### Genome sequencing, assembly, and annotation

To investigate *w*Turt’s CI-inducing ability, we obtained the *Wolbachia* genome from the *T. urticae* genome project (PRJNA914488). A total of 241.3 Gb of raw reads were generated from whole-genome sequencing on the PacBio Sequel II platform (Berry Genomics, Beijing, China). The genome was assembled using Hifiasm 0.16.1-r375 [38] and annotated with the NCBI Prokaryotic Genome Annotation Pipeline (PGAP) [39]. The proteome was functionally characterized with eggNOG-mapper v2 [40]. Prophage regions were predicted with PHASTER [41] and insertion sequences were identified with ISfinder [42].

### Identification of cif proteins in *w*Turt

Candidate CifA and CifB proteins in *w*Turt were identified by searching for similar proteins with known CifA and PD-(D/E)XK domain of CifB proteins with blastp 2.15.1+ [43]. The phylogeny of Cif protein sequences was constructed using IQ-TREE. Following Lindsey’s method [44], potential protein domains of the *cif* genes were further searched against SCOPe70 version 2.08, COG/KOG version 1.0, Pfam-A version 36.0 and SMART version 6.0 databases using HHpred web server (https://toolkit.tuebingen.mpg.de/tools/hhpred; [45]) with default parameters.

### Comparative genomics of *Wolbachia* in different *T. urticae* populations

To explore differences in the *cif* genes among *Wolbachia* strains, we sequenced genomes from four *T. urticae* populations and analyzed SNPs in *Wolbachia*. For each population, 8–10 mites were pooled into one sample, and genomic DNA was extracted using the DNeasy Blood & Tissue Kit (Qiagen). Paired-end 150 bp libraries were constructed using the TruSeq Nano DNA Library Prep Kit (Illumina), followed by whole-genome sequencing on the Illumina NovaSeq Xplus platform. Raw reads were filtered using BBtools to remove low-quality sequences, then mapped to the *Wolbachia* (*w*Turt) genome (CP114181) with BWA, sorted with SAMtools, and processed with GATK to mark PCR duplicates. Variant calling and genotyping were performed with GATK’s HaplotypeCaller and GenotypeGVCFs. High-confidence variants were selected through hard filtering based on previously reported parameters [46]. More details are provided in the “Supplementary Materials and Methods”.

### *Wolbachia* density and *cif* gene expression quantification

RNA-seq datasets of multiple developmental stages of *T. urticae*, including egg, nymph, and female adults, were downloaded from NCBI SRA database and trimmed as described before [47]. Trimmed clean reads were mapped to the transcripts predicted by *w*Turt genome (CP114181) using Salmon v. 0.14.0 [48]. The expression levels of *cif* genes are represented using TPM (Transcripts Per Million) and displayed with box plots.

For comparative expression analysis of *Wolbachia* genes using qPCR, total RNA was extracted from pools of approximately 30 spider mites per sample using RNAiso Plus (Takara Bio, Dalian, Liaoning, China) and reverse‑transcribed into cDNA with HiScript II Q RT SuperMix for qPCR (+gDNA wiper) (Vazyme Biotech). Depending on the experimental design, either *rps18* (ribosomal protein S18; tetur01g03850) or *wsp* served as the internal control. Relative gene expression levels were calculated using the 2^−ΔCT^ method. The *Wolbachia* density was quantified by qPCR using mite DNA samples. Total DNA was extracted from spider mites with the FastPure Blood/Cell/Tissue/Bacteria DNA Isolation Mini Kit (Vazyme Biotech). Copy numbers of the *Wolbachia wsp* gene and the mite single‑copy reference gene *rps18* were determined by qPCR. *Wolbachia* density was expressed as the ratio of *wsp* to *rps18* copy numbers. All qPCR reactions were performed with ChamQ SYBR qPCR Master Mix (Vazyme Biotech) on a QuantStudio 6 Flex Real‑Time PCR System (Applied Biosystems, Waltham, MA, USA) at the Public Experimental Facility, College of Plant Protection, Nanjing Agricultural University. Each sample was run in duplicate technical repeats. Differences in density and expression data among treatment groups were assessed by one‑way ANOVA. Primer sequences are listed in Table S2.

### Yeast two-hybrid assay

To investigate the interaction between CifA and CifB proteins, we used the yeast two-hybrid (Y2H) system. The full-length CifA gene was cloned into the pGBKT7 vector to create the bait vector pGBKT7-CifA. The PD-(D/E)XK domain (1–767 aa) of CifB2 was cloned into the pGADT7 vector to create the prey vector pGADT7-CifB. Both vectors were co-transformed into the Y2H Gold yeast strain (Zoman Biotech, Beijing, China). Transformants were selected on double dropout (SD-Leu/-Trp) medium. Positive interactions were confirmed by growing colonies on more stringent media: triple dropout (SD-Leu/-Trp/-His) (TDO) and quadruple dropout (SD-Leu/-Trp/-His/-Ade) (QDO) media, supplemented with X-α-gal and Aureobasidin A (AbA). Negative controls were performed with empty vectors. Colonies that grew on QDO medium and turned blue indicated a strong interaction, while blue colonies on TDO medium suggested a weak interaction. No growth or color change indicated no interaction.

**Table 1:** Statistics of crossbreeding experiment results of different mating combinations.

**Backcross**	**Treatment**	**♀ × ♂**	**Number of replicates**	**Egg number, mean ± SE**	**Hatch rate (%), mean ± SE**	**Male % of total, mean ± SE**	**CI integrity level %**
**No**	Control	JN^JN^ × JN^JN^	30	39.9 ± 1.46^a^	98.11 ± 0.44^a^	22.69 ± 1.24^a^	\
		NJ^NJ^ × NJ^NJ^	24	35.46 ± 1.55^ab^	96.3 ± 0.70^a^	14.86 ± 0.85^c^	\
		TA^TA^ × TA^TA^	34	34.88 ± 1.02^b^	97.22 ± 0.49^a^	19.30 ± 1.32^b^	\
		YLA^YLA^ × YLA^YLA^	40	33.45 ± 1.43^b^	96.51 ± 0.65^a^	24.22 ± 2.05^a^	\
		YLP^YLP^ × YLP^YLP^	27	31.70 ± 1.53^b^	97.59 ± 0.53^a^	19.11 ± 1.50^b^	\
	CI	NM^−^ × JN^JN^	24	43 ± 0.96^a^	60.68 ± 1.76^c^	29.83 ± 1.56^b^	43.86
		NM^−^ × NJ^NJ^	28	34.89 ± 0.92^b^	74.62 ± 1.85^ab^	29.11 ± 2.07^b^	35.47
		NM^−^ × TA^TA^	30	32.27 ± 1.27^bc^	73.80 ± 2.53^ab^	34.27 ± 2.41^b^	38.17
		NM^−^ × YLA^YLA^	40	30.98 ± 1.18^c^	75.22 ± 2.97^a^	49.50 ± 3.56^a^	48.06
		NM^−^ × YLP^YLP^	23	31.65 ± 1.35^bc^	65.03 ± 3.42^bc^	31.13 ± 2.48^b^	43.27
**Yes**	Control	NM^JN^ × NM^JN^	26	39.31 ± 1.34^a^	97.60 ± 0.94^a^	18.5 ± 0.78^bc^	\
		NM^NJ^ × NM^NJ^	24	30.50 ± 1.92^b^	93.53 ± 1.43^b^	19.98 ± 1.62^ab^	\
		NM^TA^ × NM^TA^	24	23.00 ± 1.00^c^	85.87 ± 5.77^ab^	23.86 ± 1.63^a^	\
		NM^YLA^ × NM^YLA^	26	23.00 ± 1.12^c^	79.71 ± 2.53^c^	16.62 ± 2.35^bc^	\
		NM^YLP^ × NM^YLP^	24	32.46 ± 1.55^b^	90.66 ± 1.67^b^	16.78 ± 1.75^bc^	\
		NM^NM^ × NM^NM^	20	30.25 ± 1.63^b^	89.95 ± 3.25^b^	16.32 ± 3.16^c^	\
	CI	NM^−^ × NM^JN^	42	30.29 ± 1.14^bc^	59.54 ± 2.67^a^	44.05 ± 3.29^b^	58.12
		NM^−^ × NM^NJ^	26	31.31 ± 2.08^ab^	51.63 ± 3.62^b^	44.57 ± 3.80^b^	61.77
		NM^−^ × NM^TA^	30	32.10 ± 1.19^ab^	52.45 ± 2.60^ab^	46.30 ± 3.25^b^	66.37
		NM- × NM^YLA^	28	26.43 ± 1.53^c^	40.97 ± 2.32^c^	82.55 ± 2.49^a^	56.93
		NM^−^ × NM^YLP^	23	32.96 ± 1.32^ab^	50.34 ± 2.73^ab^	48.46 ± 2.18^b^	89.24
		NM^−^ × NM^NM^	20	36.35 ± 1.91^a^	48.22 ± 2.94^bc^	47.51 ± 5.65^b^	65.61

Note: Different letters indicate significant difference levels of *P* < .05 calculated by Kruskal–Wallis test with Bonferroni correction for different populations.

### Yeast expression toxicity assay

The *cif* gene sequences were amplified using KOD One™ PCR Master Mix (Toyobo, Osaka, Japan) and primers listed in Table S2. The amplified fragments of *cifA* and *cifB* genes were separately cloned into the pRS425GAL1 (LEU2) and pRS416GAL1 (URA3)/pYES2 vectors using the ClonExpress II One Step Cloning Kit (Vazyme) to enable galactose-inducible protein expression in yeast [17]. To evaluate whether the lack of toxicity was associated with inefficient heterologous expression, a yeast codon-optimized sequence encoding the full CifA2 and PD-(D/E)XK nuclease domain of CifB2 was commercially synthesized. The codon-optimized fragment was cloned into the pRS425GAL1 and pRS416GAL1 vectors, respectively, using the same cloning strategy described above. The indicated plasmids were transformed into the MHY1774 (W303-1A) yeast strain. For growth testing, transformants were cultured at 30°C for 2 days in synthetic defined (SD) medium supplemented with raffinose and lacking uracil (Ura) and leucine (Leu). Cultures were then subjected to 5-fold serial dilution and spotted onto SD plates lacking Ura and Leu and containing either 2% galactose or glucose as the carbon source. Plates were incubated at 30°C and 36°C for 48–96 h, and yeast growth was monitored during this period.

### Transgenic *Drosophila melanogaster* experiment

The *cifA* and *cifB* genes were amplified from *T. urticae* DNA and cloned into the pJFRC28 vector to create UAS-cifA and UAS-cifB (partial cifB) plasmids. These plasmids were injected into *Drosophila* attP2 embryos using the PhiC31 integrase system. The injected embryos were cultured at 25°C. P0 adults were crossed with w^1118^ flies, and F1 miniwhite^+^ individuals were backcrossed with TM2/TM6B to stabilize the transgenes. For germline expression, UAS-cif/TM6B lines were crossed with Nos-gal4/CyO flies, and F2 progeny were intercrossed to establish stable lines. Transgene integration was confirmed by PCR.

Mating experiments were performed with different *Drosophila* lines in culture tubes, with two males and one unmated female per tube. After 2 days, the number of eggs laid was counted, and the larvae were separated to calculate the hatching rate. Each cross was repeated 20 times. Further details are available in the “Supplementary Materials and Methods”.

## Results

### *Wolbachia* of different *T. urticae* geographic populations induces CI

To investigate the influence of *Wolbachia* from different wild *T. urticae* populations, we examined the fecundity, hatching rates, and sex ratios of mites collected from various field sites. Significant variation was observed in the number of eggs laid per female across the five natural populations in both Control and CI crosses (Kruskal–Wallis test, *P* < .01) (Table 1). In Control crosses, no significant differences were found in hatching rates among the five populations (Kruskal–Wallis test, *P* > .05), but in CI crosses, significant variations appeared (Kruskal–Wallis test, *P* < .01) (Table 1). Furthermore, the sex ratios differed significantly among the five populations in both Control and CI crosses (Kruskal–Wallis test, *P* < .01).

When comparing the CI and Control crosses, no significant difference in fecundity was found within each population (Wilcoxon test, *P* > .05) (Fig. 1A). However, hatching rates were significantly lower in CI crosses compared to Control crosses across all populations (Wilcoxon test, *P* < .0001). In Control crosses, the hatching rates were consistently above 96%, while in CI crosses, 25% to 40% of eggs failed to hatch (Fig. 1B). The male proportion in CI crosses was significantly higher than in Control crosses for all populations (Wilcoxon test, *P* < .01) (Fig. 1C). Regarding CI intensity, the YLA population demonstrated the highest CI intensity at 47.35%, with the remaining populations showing CI strengths between 34% and 40% (Fig. 1G, Table 1). These results clearly demonstrate that *Wolbachia* from different *T. urticae* populations induces varying levels of CI.

**Figure 1:**
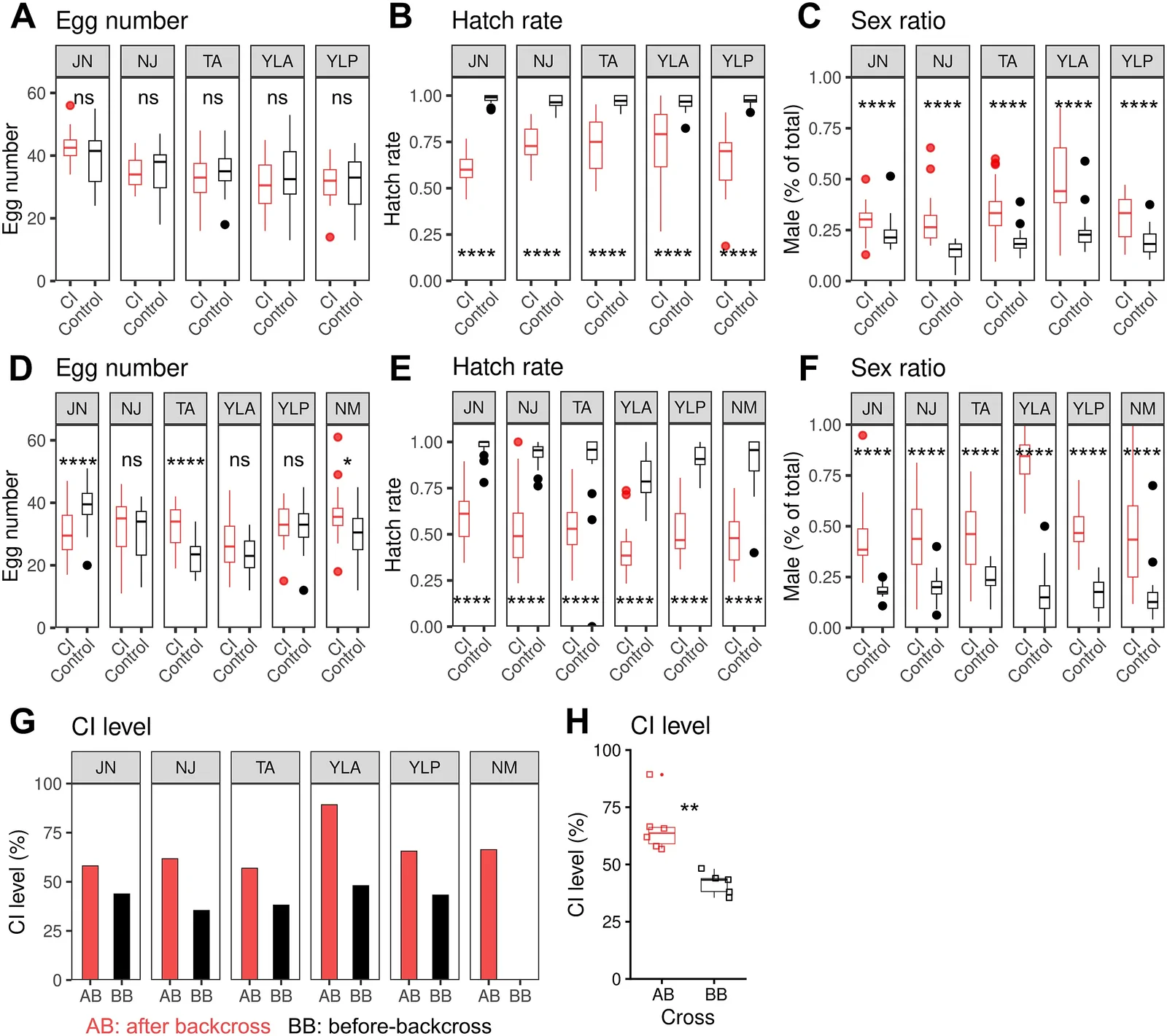
Various effects of *Wolbachia* on the reproduction of *Tetranychus urticae* before and after unifying the nuclear genetic background. (A) Number of deposited eggs, (B) hatch rate, and (C) rate of hatched male offspring in different *T. urticae* populations (JN, NJ, TA, YLA, YLP). The control group (Control) consisted of *Wolbachia*-infected female and male mating; the CI group consisted of *Wolbachia*-uninfected female and infected male mating. (D) Number of deposited eggs, (E) hatch rate, and (F) rate of hatched male offspring in *T. urticae* populations carrying different *Wolbachia* strains, with the unified nuclear genetic background (all NM population nuclear genes). The control and CI groups were set up as in (A–C). (G) CI levels in different *T. urticae* populations. (H) Direct comparison of CI levels between backcrossed populations (with unified nuclear genetic background) and non-backcrossed populations (with non-unified nuclear genetic background). All data were analyzed using the Wilcoxon test. Significance annotations: ns, no significant difference (*P* ≥ .05); ^****^, *P* < .0001.

### *Wolbachia*-induced CI varies among *T. urticae* populations with identical nuclear genetic background

To examine how *Wolbachia* strains from different *T. urticae* populations influence CI, we conducted backcrossing experiments to standardize their nuclear genetic backgrounds to a common background (NM) (Fig. S1). Despite this, fecundity remained significantly different across populations in both Control and CI crosses (Kruskal–Wallis test, *P* < .01, Table 1). Hatching rates in CI crosses were significantly lower than in Control crosses across all populations (Wilcox test, *P* < .01) (Fig. 1E), with the NM^NJ^ population showing a 44% reduction in hatch rate (Wilcox test, *W* = 24.5, *P* < .01). Additionally, the proportion of males in the CI cross of the NM^NJ^ population increased by ~24.6% compared to the Control cross (Wilcox test, *W* = 397.5, *P* < .01) (Fig. 1F). These results confirm that *Wolbachia* strains within each population induce significant CI.

Further analysis revealed that the TA^TA^ population exhibited significantly higher fecundity than the NM^TA^ population in Control crosses (Wilcox test, *W* = 45.5, *P* < .01) (Fig. 1D), and the YLA^YLA^ population had a higher hatching rate compared to the NM^YLA^ population (Wilcox test, *W* = 104.5, *P* < .01) (Fig. 1E). The sex ratio of the NJ^NJ^ population was significantly lower than the NM^NJ^ population (Wilcox test, *W* = 376, *P* < .01) (Fig. 1F, Table 1). After genome homogenization, CI intensity generally increased across populations (Fig. 1G and H), suggesting that nuclear genetic background plays a significant role in fecundity, hatching rates, and sex ratios in *T. urticae*. Notably, the NM^JN^ population produced the highest number of eggs per female in Control crosses (Fig. 1A), and the NM^YLA^ population exhibited the lowest hatching rates across both Control and CI crosses (Fig. 1E). In CI combinations, the NM^YLA^ population had a significantly higher male proportion, which also correlated with the highest CI intensity observed in this population (Fig. 1F and G). These findings highlight that *Wolbachia*-induced CI varies among *T. urticae* populations, even when the hosts share the same nuclear genetic background.

### Identifying CI factor genes (*cif*) from *w*Turt genome

To investigate why *Wolbachia* in *T. urticae* (*w*Turt) induced CI, we sequenced its complete genome. The *w*Turt genome is 1.65 Mb in length with a GC content of 34.04%. Annotation using the PGAP pipeline revealed 1744 genes, including 43 RNA genes and 1701 protein-coding sequences (1306 CDSs and 395 pseudogenes). Two adjacent genes encoding proteins CifA (CI factor A, CidA, or CinA) and CifB (CI factor B, CidB, or CinB) may be the key factors underlying *Wolbachia*-induced CI in insects [15, 16, 19]. We identified two tandem pairs of *cifA-cifB* genes from the *w*Turt genome (Fig. 2). Phylogenetic analysis revealed that the two Cif protein sequences are classified as Type V (Fig. 2). The first pair, O1Q06_06310 (CifA1) and O1Q06_06330 (CifB1), has close homologs in *w*Ttru. In contrast, the second pair, O1Q06_06880 (CifA2) and O1Q06_06875 (CifB2), forms another monophyletic group (Fig. 2).

**Figure 2:**
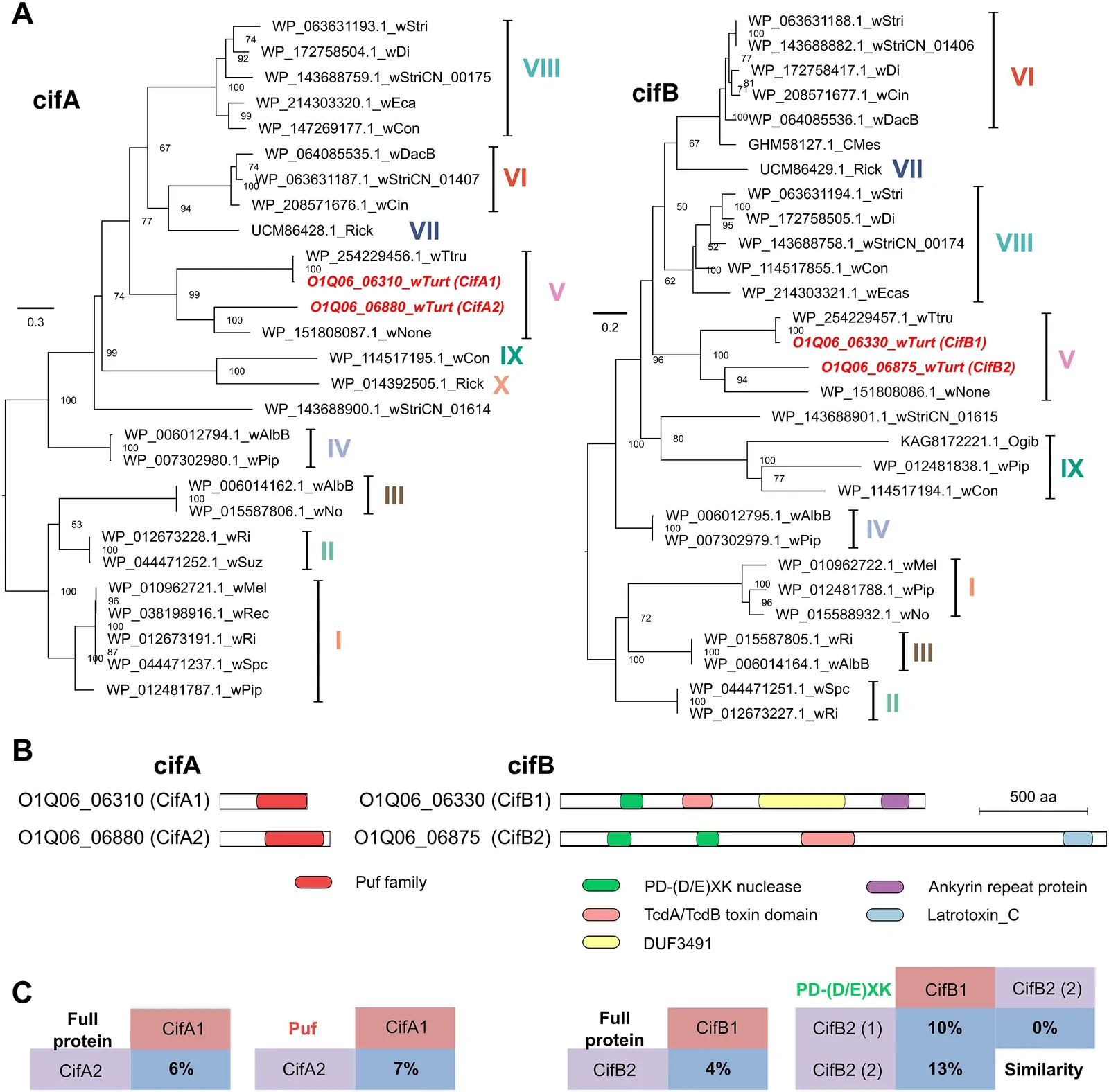
Phylogeny and predicted structures of CifA and CifB proteins. (A) Phylogenetic tree of CifA and CifB proteins. The respective NCBI accession numbers for the Cif proteins and corresponding *Wolbachia* strain names are displayed. The CifA tree was generated using the JTT + F + G4 substitution model, while the CifB tree used the cpREV + G4 model. The scale bar indicates the average substitutions per site. Nodes with bootstrap support >50% are labeled, and clades are grouped by colored vertical bars. (B) Predicted structures of the two pairs of CifA and CifB proteins in *w*Turt. (C) Amino acid similarity of the full proteins and key domains of CifA and CifB.

In the *w*Turt genome, both CifA proteins possess a Puf family RNA-binding domain, a feature common to all known CifA types (Fig. 2B). It should be noted that the crystal structures of CinA and CidA proteins revealed that this domain actually comprises a series of HEAT repeats, a structure related to but distinct from the Puf domain [49]. However, the STE-like transcription factor domain, characteristic of CifA types I to IV, is absent in *w*Turt’s CifA proteins (Fig. S2). Similar to those in *w*StriCN [23], both CifB1 and CifB2 are significantly longer than CifB proteins of Types I-IV (Fig. S2). CifB2 is predicted to contain two active PD-(D/E)XK endonuclease NucS domains (Fig. 2B), a trait shared with other CifB types, whereas CifB1 has only one PD-(D/E)XK domain. Additionally, CifB1 includes a TcdA_TcdB_pore domain, a DUF3491 domain of unknown function, and an ANK domain. CifB2, on the other hand, contains a TcdA_TcdB_pore domain and a latrotoxin_C domain. Notably, the shared domains between the two CifA-CifB pairs exhibit low sequence similarity (Fig. 2C); for instance, the Puf domains of CifA1 and CifA2 share only 7% aa similarity, and the three PD-(D/E)XK domains share less than 15% aa similarity. In addition, we identified the typical active site residues (E…D…EXK…Q) of the fourth REase domain [22] from the detected PD-(D/E)XK domain of cifB1 and the first PD-(D/E)XK domain of cifB2 (Fig. S3).

PHASTER identified two prophage regions (WOTurt1 and WOTurt2) in the *w*Turt genome, with a combined size of 16.6 kb (Table S3). However, neither *cifA-cifB* gene pair is located within PHASTER-predicted WO regions. Both *cifA-cifB* operons were surrounded by IS630 or IS982 family transposases on *w*Turt genome. Unlike the *cifA2-cifB2* pair, *cifA1* and *cifB1* are not adjacent but are separated by a transposase (ISWpi16 member) and two genes of unknown function (annotated as hypothetical proteins). Furthermore, multiple transposase-encoding genes flank both *cifA1* and *cifB1* (Fig. 3A), suggesting that this gene pair may have been horizontally transferred from somewhere else. From the RNA-seq dataset, we found that the expression of *cifA2* and *cifB2* genes was higher than that of *cifA1* and *cifB1* across various stages of *T. urticae* (Fig. 3B). Moreover, cifA1 seemed not to be expressed at all. This suggests that *cifA2* and *cifB2* may play a more prominent role.

**Figure 3:**
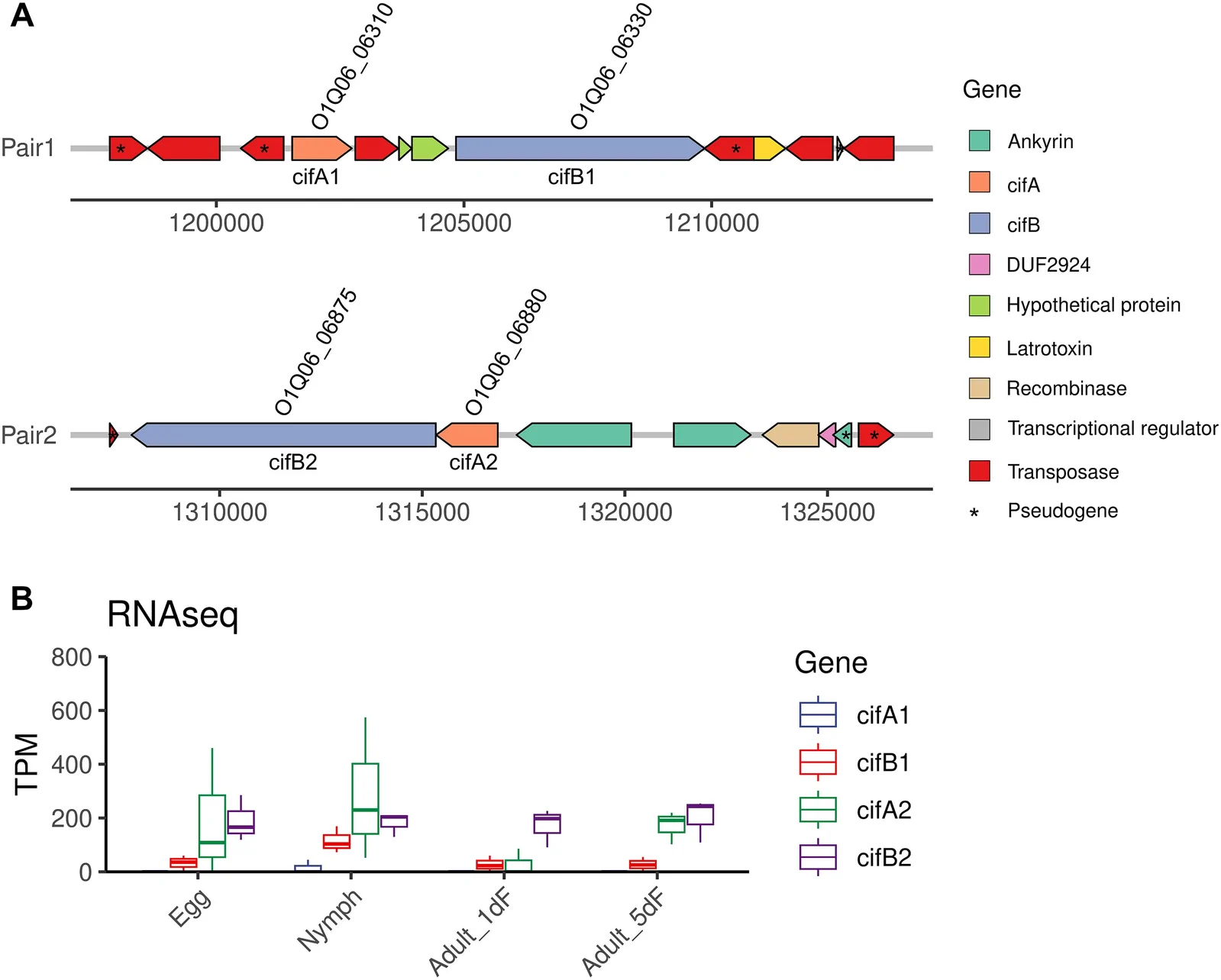
Genetic architecture and expression dynamics of two *cifA/cifB* gene pairs (Pair1 and Pair2) in *w*Turt. (A) The genomic organization of the two loci. Pair1 (coordinates 1 200 000–1 210 000) contains *cifA1* (O1Q06_06310) and *cifB1* (O1Q06_06330) flanked by transposase and latrotoxin genes, while Pair2 (1310000–1 325 000) includes *cifB2* (O1Q06_06875) and *cifA2* (O1Q06_06880) adjacent to ankyrin and recombinase genes. (B) RNAseq expression profiles of two *cifA/cifB* gene pairs across developmental stages of *T. urticae*. Adult_1dF indicates 1-day old female adult.

### Genomic variations of *w*Turt from different *T. urticae* populations

Whole-genome sequencing and variant calling revealed 143 genetic variants across *Wolbachia* genomes from different geographic populations, comprising 96 SNPs, 28 insertions, and 19 deletions. Among these, 14 variants with HIGH or MODERATE functional impact were identified in protein-coding genes, including hypothetical proteins (O1Q06_03155, O1Q06_00650), *mutL* (DNA mismatch repair endonuclease), *ftsZ* (cell division protein), *sdhC* (succinate dehydrogenase cytochrome b556 subunit), and O1Q06_03055 (twin-arginine translocase TatA/TatE family subunit) (Table S4). However, no SNPs were detected within the *cif* operon regions, suggesting that the *Wolbachai cif* genes in tested spider mite populations are the same at the genomic level (Fig. S4).

We examined the expression of the two *cif* gene pairs (*cifA1-cifB1* and *cifA2-cifB2*) in *w*Turt from different *T. urticae* populations using RT-qPCR. The expression of the *Wolbachia* surface protein (*wsp*) gene varied across populations (Fig. 4A, Fig. S5A), suggesting differences in *Wolbachia* density. This density was positively correlated between males and females (Fig. S5B), showing that the genetic background of *T. urticae* populations influences *Wolbachia* density. As seen in previous transcriptomic data, *cifA1* and *cifB1* had very low expression in both males and females (Fig. 4B, Fig. S5C), with no correlation between their levels (*P* > .05) (Fig. 4C, Fig. S5D). However, *cifA2* and *cifB2* showed high expression, with significant variation across populations (Fig. 4B, Fig. S5C), and their expression was strongly linked (Fig. 4D, Fig. S5D). The correlation slope was nearly the same for *Wolbachia* from both male and female (0.97 for males, 1.1 for females), suggesting they share a common promoter. Furthermore, the expression of *cifB2* per *Wolbachia* cell stayed stable even as *Wolbachia* density increased (*P* > .05 for both males and females) (Fig. S5E), indicating that its expression is mainly determined by *Wolbachia* density (Fig. 4E).

**Figure 4:**
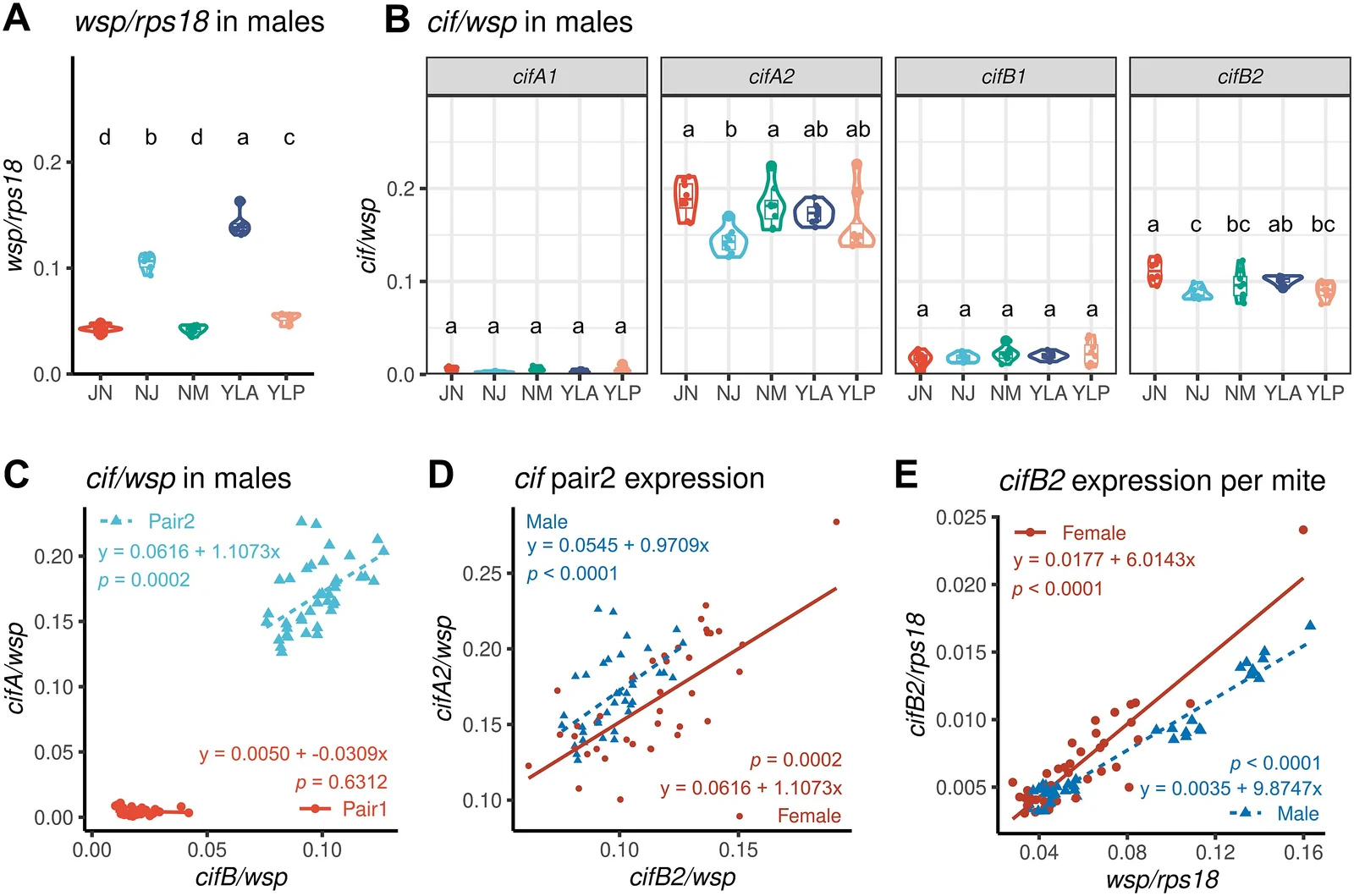
Expression patterns and correlation analysis of *wsp* and *cif*-related genes of *w*Turt across different *T. urticae* populations. (A) Expression levels of the *Wolbachia wsp* gene in male spider mites from different populations, normalized as *wsp/rps18*. (B) Expression levels of each *cif* gene in male *T. urticae* infected with *w*Turt, normalized as *cif/wsp*. Different letters indicate significant statistical differences in one-way ANOVA analysis (*P* < .05). (C) Correlation analysis between the expression of *cifA* and *cifB* in males (*cif/wsp*). Pair1 (*cifA1/cifB1*) is represented by red circles, and Pair2 (*cifA2/cifB2*) is represented by blue triangles. (D) Correlation analysis of *cifA2* and *cifB2* expression in males and females, normalized as *cif/wsp*. Males are represented by blue triangles, and females by red circles. (E) Correlation analysis of *cifA2* and *cifB2* expression in males and females, normalized as *cif/rps18*. Males are represented by blue triangles, and females by red circles. The regression equations and *p* values for correlation analyses are displayed in the figure.

### Interaction analysis of CifA^*w*Turt^ and CifB^*w*Turt^

It has been shown that CifA interacts with CifB to prevent CifB-induced embryonic death [15, 17]. We expressed CifA in prokaryotic (*E. coli*) expression systems, but it only formed inclusion bodies (data not shown), preventing pulldown experiments to test the interaction. Therefore, we used a yeast two-hybrid system to check the CifA-CifB interaction. No colonies were observed in the BD-CifA1 + AD or BD-CifA2 + AD groups on triple dropout medium (TDO, SD-Leu/-Trp/-His), confirming no self-activation. Additionally, BD-CifA1 + AD-CifB1 did not produce blue colonies at any dilution on QDO plates, suggesting little or no interaction between CifA1 and CifB1; weak growth on the selection plates suggested a potential weak interaction or poor expression of one or both proteins. However, BD-CifA2 + AD-CifB2(PDDEXK: 1–767 aa) showed normal colony growth and blue coloration at dilutions of 10^−1^ to 10^−4^, suggesting binding between CifA2 and CifB2(PDDEXK) (Fig. 5A).

**Figure 5:**
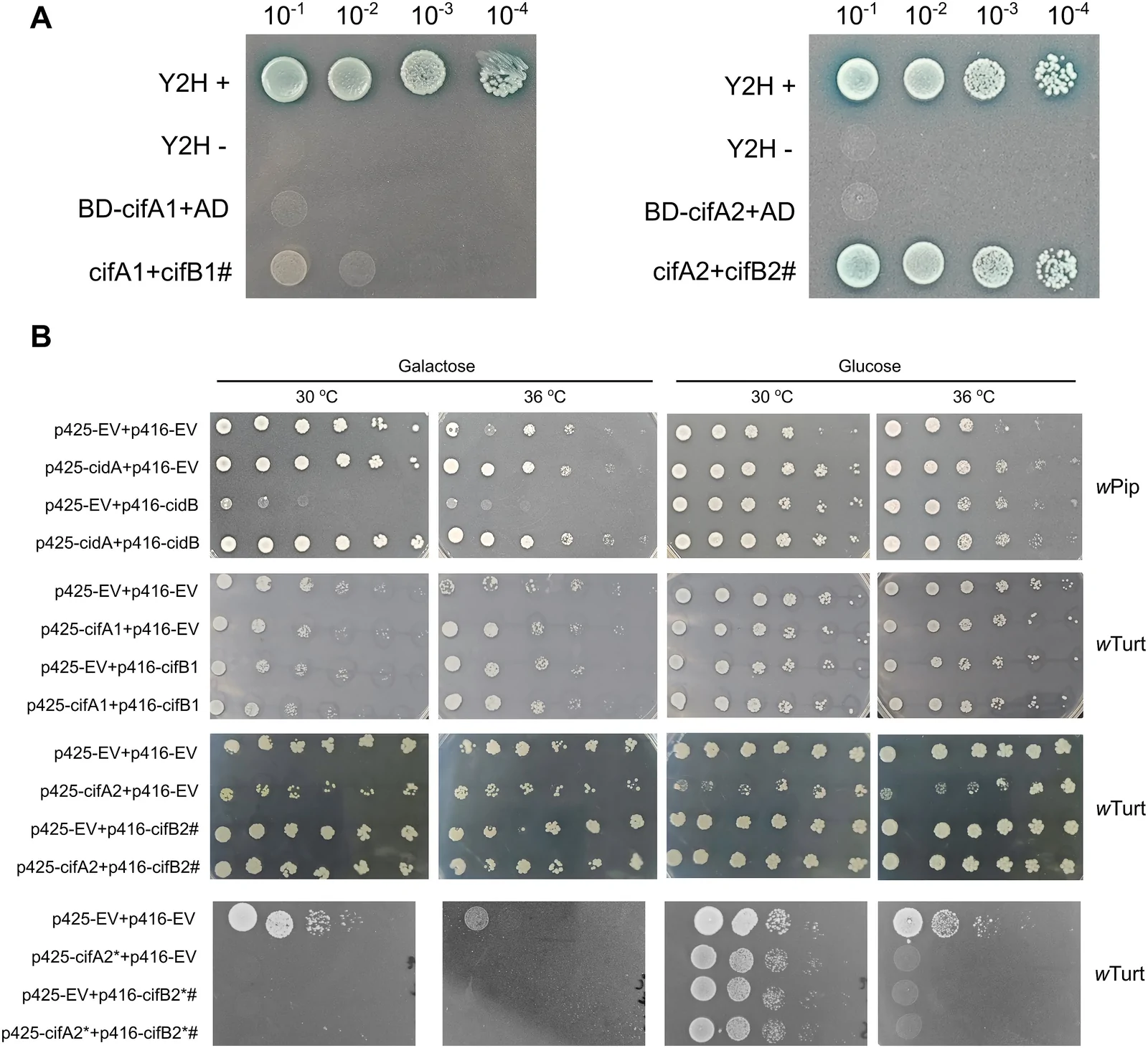
Interaction between CifA and CifB and yeast toxicity growth analysis. (A) Yeast two-hybrid assay (Y2H) testing the interaction between CifA and CifB pairs from *w*Turt. Yeast cells co-transformed with BD-CifA and AD-CifB were serially diluted (10^−1^ to 10^−4^) and grown on Y2H+ selective medium (QDO plates). Pair 1 (CifA1 + CifB1) showed no colony growth (negative interaction), while Pair 2 (CifA2 + CifB2_PDDEXK:1–767 aa) formed blue colonies (positive interaction). Y2H- non-selective medium was used to show background growth. (B) Yeast toxicity growth analysis of Cif proteins. *Saccharomyces cerevisiae* cells expressing *w*Turt *cifA* and *cifB* alleles were transformed with pRS425GAL1 and pRS416GAL1 plasmids, inducing expression under the galactose-inducible GAL1 promoter. Yeast cells were serially diluted, spotted on selective plates with different carbon sources, and incubated at 30°C and 36°C for 2.5 days. The # next to cifB indicates that ​CifB consists solely of the PD-(D/E)XK domain. The ^*^ next to cif indicates that the codon was optimized. EV indicates empty vector; *cif* genes from *w*Pip were used as a positive control.

### Analysis of CifA^*w*Turt^ and CifB^*w*Turt^ toxicity in yeast

Yeast growth assays have been widely used as a heterologous readout of CifB toxicity and CifA-mediated rescue in studies of *Wolbachia*-induced CI [15, 17]. Because repeated attempts to clone and express full-length *cifB2* in yeast were unsuccessful, we focused on the predicted PD-(D/E)XK nuclease domain of CifB2^*w*Turt^. Expression of CidB^*w*Pip^ consistently caused growth defects at higher temperatures (Fig. 5B), validating the assay system. In contrast, native CifB1^*w*Turt^ and CifB2(PDDEXK: 1–767) ^*w*Turt^ did not induce detectable growth defects under galactose induction (Figs 5B and S6). Co-expression with the corresponding CifA did not exacerbate or alleviate these growth defects. To address whether the absence of toxicity reflected poor heterologous expression, we further generated a yeast codon-optimized version of the CifB2 PD-(D/E)XK domain. This codon-optimized construct showed induction-dependent toxicity at 30°C, as yeast grew on glucose but failed to grow on galactose. However, yeast growth was impaired even under repressing glucose conditions at 36°C, and co-expression of CifA2 failed to rescue the toxicity of CifB. CifA2 alone also caused growth defects under inducing or high-temperature conditions (Fig. 5B). These results suggest that codon optimization can unmask toxicity of the CifB2 nuclease domain in yeast, but the assay did not validate a canonical CifA-CifB toxin-antidote mechanism for *w*Turt.

### Assessing the CI-inducing capacity of CifA^*w*Turt^ and CifB^*w*Turt^ in *D. melanogaster*

By constructing multiple transgenic *D. melanogaster* lines carrying the full-length cifA sequence, as well as specifically splitting the cifB gene by domain (the cloning of full length cifB1 and cifB2 was unsuccessful because of too large gene sequence), we systematically conducted genetic crosses to verify whether the CI factors encoded by *w*Turt could induce CI in insects (Fig. S7). We compared egg production and hatching rates across different fly mating combinations. The results showed no statistically significant differences in egg production (Kruskal–Wallis test, *W* = 4.33, *P* = .93). Similarly, no significant differences were observed in hatching rates between the mating combinations (Kruskal–Wallis test, *W* = 2.46, *P* = .99). These findings imply that the expression of *cifA1, cifB1, cifA2,* and *cifB2*, either individually or in combination, fails to induce CI in *D. melanogaster* (Fig. 6).

**Figure 6:**
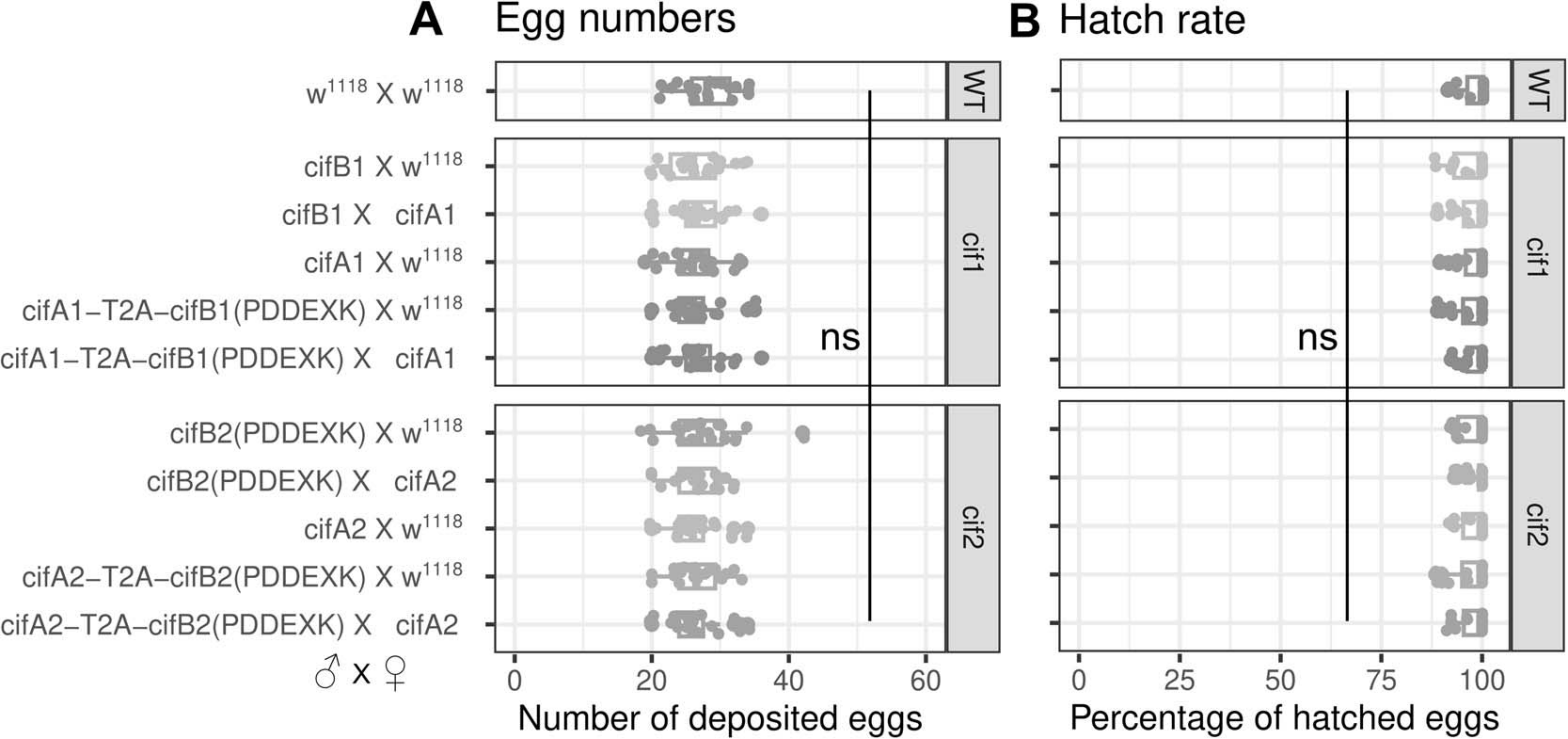
CI induction by *w*Turt cif gene pairs in *Drosophila* system. (A) Number of deposited eggs per female and (B) Egg hatch rate among different transgenic *D. melanogaster* mating combinations. No significant differences were observed across all groups (ns, *P* > .05). w^1118^ is the wild-type control. T2A is the co-expression linker; cifB1(PDDEXK) and cifB2(PDDEXK) correspond to the 1–380 aa of cifB1 and 1–767 aa of cifB2 functional domains, respectively. Data distribution is visualized using boxplots. Each dot represents the results of a 1 × 1 pair of flies. Data were analyzed using ANOVA followed by Tukey’s post hoc test.

## Discussion

This study advances our understanding of the mechanism behind *Wolbachia*-induced CI in *T. urticae* spider mites. *Wolbachia*-induced CI exhibits significant variability both within and between *T. urticae* populations. Consistent with earlier findings [50, 51], we observed varying levels of *Wolbachia*-mediated incompatibility across *T. urticae* populations. These results clearly highlight the widespread occurrence of *Wolbachia*-induced CI in different mite populations.

Previous studies have shown that *Wolbachia*-induced CI in *T. urticae* is influenced by both the *Wolbachia* strain and the host genetic background. Suh *et al.* [52] suggested that the phenotypic effects of *Wolbachia* might be linked to genetic diversity and host plant associations in Korean populations. Wybouw *et al.* [53] observed CI variation when a single CI-inducing *Wolbachia* strain was transferred to mites with different host genetic backgrounds. Our previous work [35] also found that mites with the same *Wolbachia* strain but different host genetics exhibited varying CI levels. Additionally, mites with different *Wolbachia* strains but the same host background showed differences in CI strength (Table 1). These findings suggest that while host genetic background can influence CI, the *Wolbachia* strain plays a key role in determining CI strength in *T. urticae* populations.

The main bacterial factors that induce CI in *Wolbachia* are the CifA and CifB proteins. These proteins are likely encoded by two adjacent genes in either *Wolbachia* or its associated prophage genomes [3]. In the *w*Turt genome, we have identified two pairs of *cif* genes (*cifA1-cifB1* and *cifA2-cifB2*). Some studies have reported that the number of *cif* genes encoded by *Wolbachia* is related to the strength of the CI it induces [16], and the CI variations are related to *Wolbachia cif* gene divergence in *Culex pipiens* [54]. Although the CI induced by *w*Turt in *T. urticae* is incomplete, its intensity is higher than that induced by *w*Ttru in *T. truncatus* [55]. However, we don’t think this difference is likely to stem from *w*Turt encoding two Cif protein pairs versus one in *w*Ttru, since one of the pairs appears to be non-functional. The reason remains unclear and requires further investigation.

Phylogenetic analysis shows that the two pairs of *cif* genes in *w*Turt belong to type V, with CifB1 and CifB2 being notably longer than other cifB proteins. These pairs exhibit less than 10% amino acid sequence similarity, suggesting independent evolutionary origins rather than gene duplication (Fig. 2). Both pairs contain Puf/HEAT domains, with CifA2 having a longer Puf domain than CifA1. CifB1 has one putatively active PD-(D/E)XK domain, while CifB2 has two, along with TcdA/TcdB toxin and latrotoxin C domains. The Puf and PD-(D/E)XK domains are essential for the proper function of CifA and CifB [56, 57]. TcdA/TcdB is a pore-forming toxin that may enhance bacterial virulence [58]. Latrotoxin, also found in *Wolbachia*-associated WO phage [59], correlates with *Wolbachia* virulence in *D. melanogaster*, suggesting a potential link to pathogen virulence [60].

We found that pair 2 of the *cif* genes from *w*Turt in *T. urticae* was expressed significantly more than pair 1. Yeast two-hybrid experiments indicated that CifA2 and the PD-(D/E)XK domain of CifB2 interacted, which is the first demonstration of a protein–protein interaction for a type V CifA-CifB system and suggests their possible involvement in the rescue effect [61]. Pair 1 was nearly undetectable in both laboratory and wild WI *T. urticae* populations, indicating potential degeneration into pseudogenes. This suggests that pair 2 plays a key role in *w*Turt-induced CI. Although no SNP differences were found in *cif* genes across *T. urticae* populations, expression levels of pair 2 varied significantly, possibly explaining the CI intensity differences in *T. urticae*, as seen in *Drosophila* [62]. Moreover, cifB2 expression strongly correlated with *Wolbachia* density in *T. urticae*, suggesting that bacterial density may regulate CI intensity. However, in agreement with our observations in mites, previous studies showed no significant correlation between *Wolbachia* density and CI intensity across populations [63, 64], indicating that other factors also influence CI intensity.

The yeast system is frequently used to investigate CifB toxicity and CifA’s rescue effect [65]. Previous studies have shown that CidB/CinB proteins from *w*Pip, *w*No, and *w*Ha cause yeast growth defects and toxicity, which can be suppressed by co-expression with CidA/CinA [15–19, 63]. In our initial assays, neither CifB from pair 1 nor pair 2 of *w*Turt induced toxicity in yeast. While this suggests a lack of toxic effect, several limitations must be acknowledged: (i) we did not confirm expression at the protein or RNA level, and (ii) our toxicity assays used truncated CifB constructs containing only the PD-(D/E)XK nuclease domains, which may not fully recapitulate the function of the complete protein. Consistent with this concern, codon optimization of the CifB2 PD-(D/E)XK domain revealed induction-dependent toxicity at 30°C, suggesting that the native construct may have been poorly expressed in yeast. However, this toxicity was not rescued by CifA2, and the optimized construct also showed non-canonical behavior at 36°C, where growth was impaired even under repressing glucose conditions. CifA2 alone also caused growth defects under inducing or high-temperature conditions. Thus, while codon optimization indicates that the CifB2 nuclease domain can be toxic when efficiently expressed, our yeast assays did not validate a canonical CifA-CifB toxin-antidote mechanism for *w*Turt. Notably, Beckmann *et al.* [17] reported that the isolated PD-(D/E)XK domain of Type V CifB from *w*Str (reported as *w*StriCN [23]) exhibited mild toxicity in yeast. This suggests that the yeast system is generally responsive to truncated CifBs, supporting the validity of our assay setup despite the negative results for *w*Turt.

Although *Wolbachia* cannot currently be cultured or genetically manipulated *in vitro*, transgenic expression of Cif proteins in *Drosophila* successfully induces CI, proving these effectors are sufficient for CI phenotypes [16, 57, 63]. In contrast, expression of *w*Turt Cifs in *Drosophila* did not affect embryonic hatchability, unlike strong CI-inducing strains like *w*Mel, *w*Pip, and *w*No [16, 17, 63]. This negative result may reflect several non-mutually exclusive factors. First, *w*Turt’s Cifs belong to clade V, which differs structurally from clades I–IV [23], and many type V *cif* genes have lost function or become pseudogenes [24]. Second, *w*Turt-cifB1 is not expressed and encodes only a single putative PD-(D/E)XK nuclease domain, whereas two such domains are required in CinB from *w*Pip [21]. Third, CifB2’s large size (2503 amino acids) might require full-length expression for functionality. This size complicates vector construction and may exceed expression limits in transgenic flies, possibly explaining the absence of CI in our experiments.

CI induction by certain CifB proteins may depend on host-specific cofactors, which may be absent or incompatible in heterologous hosts [66]. Previous studies show that the biochemistry of CifA and CifB and the rescue function of CifA are highly sensitive to partner binding and host context, with nuclear modifiers influencing CI strength [17, 18]. In our study, CifAs and CifBs of *w*Turt did not induce detectable CI in *D. melanogaster*. In yeast, codon optimization revealed toxicity of the CifB2 PD-(D/E)XK nuclease domain, but this toxicity was not rescued by CifA2 and showed non-canonical behavior under high-temperature conditions. These results suggest that, although *w*Turt’s CifB2 may retain intrinsic toxic potential, the canonical CifA-CifB toxin-antidote mechanism was not functionally recapitulated in heterologous systems. This indicates that the *w*Turt CI system may be mechanistically unique or strictly dependent on strain-specific cofactors. Future research should focus on more permissive models that resemble the native host to better understand the biochemical requirements of CI in clade V.

In summary, our study shows that *Wolbachia*-induced CI in *T. urticae* is shaped by symbiont strain diversity and host nuclear background, with geographic populations showing variable CI intensity even under identical genetic conditions. Genomic analysis of the *w*Turt strain identifies two Type V *cif* gene pairs, with *cifA2-B2* likely serving as the primary CI effector. The unique structure of CifB2, including dual PD-(D/E)XK nuclease domains and toxin-related regions, points to evolutionary divergence in CI effectors in spider mites. Functional assays yielded mixed evidence: codon-optimized CifB2 nuclease domain was toxic in yeast, but CifA2 failed to rescue this toxicity, and transgenic *Drosophila* assays did not reproduce CI-related phenotypes. These findings emphasize the host-specific nature of *Wolbachia* reproductive manipulation and provides a framework for future validation of Cif function in agriculturally important mite pests.

## Supplementary Material

Supplementary_material_ycag263

## Data Availability

The sequencing data of *T. urticae* wild populations generated in this study have been deposited in the NCBI BioProject database under accession number PRJNA1417448. The assembled *w*Turt genome data have been deposited in the NCBI database with the accession number CP114181.
