# Supplementary material for: Genomic stability and differential expression of type V cif genes in Wolbachia with variable cytoplasmic incompatibility phenotypes in spider mites
Source: ISME Commun. 2026 Sep 11;6(1):ycag263. doi: 10.1093/ismeco/ycag263 (PMC13657049; doi:10.1093/ismeco/ycag263)
Supplement: Supplementary file 1 [file supplementary_material_ycag263.zip › Suppl methods and figures.docx]

Supplementary Information for

**Genomic stability and differential expression of type V cif genes in *Wolbachia* with variable cytoplasmic incompatibility phenotypes in spider mites**

**Authors**

Xiao-Li Bing^1^, Shuo Gao^1^, Jia-Mei Li^1^, Lei Chen^1^, Huan Wang^1^, Yu Liu^1^, Huan-Huan Liu^1^, Xiao-Yue Hong^1 *^

^1^ Sanya Institute of Nanjing Agricultural University, State Key Laboratory of Agricultural and Forestry Biosecurity, Key Laboratory of Integrated Management of Crop Disease and Pests, Ministry of Education, Department of Entomology, College of Plant Protection, Nanjing Agricultural University, Nanjing, Jiangsu 211800, China

Running Head: *Wolbachia* induced cytoplasmic incompatibility in mites

*Corresponding author: Xiao-Yue Hong, E-mail: xyhong@njau.edu.cn, College of Plant Protection, Nanjing Agricultural University, Nanjing, Jiangsu 211800, China.

**This file includes:**

Supplementary Materials and Methods

Supplementary References

Supplementary Figures S1 to S7

Supplementary Tables S1 to S4

**Supplementary Materials and Methods**

Effect of *Wolbachia* infection on mite fecundity, hatch rate, sex ratio, and CI phenotype

A newly emerged virgin female was placed with two males for mating for two days. Then the males were removed and the female was left to lay eggs for another three days. The number of eggs laid per female, the number of hatched eggs, and the number of female and male offspring adults were recorded to calculate mite fecundity, embryonic mortality, and sex ratio (% of males among total adults). It’s noted that 1-day-old virgin males were provided to avoid potential effects of male aging or repeated, consecutive mating (Sun, et al. 2016). If a male died during mating, a new male was immediately added; if a female died during spawning, the treatment was nullified. Each cross contained at least 24 biological replicates. Following previous studies (Mouton, et al. 2005; Nguyen, et al. 2017), samples that produced fewer than ten eggs were excluded. The calculation of CI intensity was indicated on “Materials and Methods”.

Comparative genomics of *Wolbachia* in different *T. urticae* populations

To investigate potential differences in the *cif* gene among *Wolbachia* strains from different populations, we performed genome sequencing on four *T. urticae* populations and analyzed the single nucleotide polymorphisms (SNPs) in *Wolbachia*. For each population, 8-10 leaf mite individuals were selected and pooled as a single sample. Genomic DNA was extracted using the DNeasy Blood & Tissue Kit (Qiagen, Hilden, Germany) according to the manufacturer's protocol. The quality of the extracted DNA was assessed through agarose gel electrophoresis, while DNA concentration and purity were measured using a NanoDrop 2000 (Thermo Fisher Scientific, Waltham, MA, USA) and a Quantus Fluorometer (Promega, Madison, WI, USA). Next, paired-end 150 bp libraries with short inserts (~500 bp) were constructed using the TruSeq Nano DNA Library Prep Kit (Illumina, San Diego, CA, USA). Whole-genome sequencing was performed on the Illumina NovaSeq Xplus platform.

The quality of raw reads were checked and filtered using BBtools 38.42 (https://bbmap.org) to remove low-quality reads containing adapters, with average base quality scores below 20 at both ends, with more than five ambiguous bases (Ns), or with a trimmed length shorter than 15 bp. Clean reads were then mapped to the indexed genomes of *Wolbachia* (*w*Turt) using BWA 0.7.17 (Li and Durbin 2009), sorted and indexed with SAMtools 1.13 (Li, et al. 2009), and further processed with GATK 4.2.2.0 (McKenna, et al. 2010) to mark PCR duplicates. The variant sites were called and genotyped by the HaplotypeCaller and GenotypeGVCFs modules in GATK under default parameters. In order to retain high-quality and high-confidence variant sites and to provide a reliable basis for functional annotation, we performed hard filtering of the sequence data using our previously reported parameters (Chen, et al. 2025).

Transgenic *Drosophila* *melanogaster* experiment

The coding sequences of *cifA* and *cifB* were amplified from the extracted DNA of *T. urticae* NM populations using the KOD One™ PCR Master Mix (Toyobo) and cloned into pJFRC28-10XUAS-IVS-GFP-p10 vector via homologous recombination (ClonExpress Ultra One Step Cloning Kit, Vazyme), generating UAS-*cifA* and UAS-*cifB* plasmids. Plasmids (500 ng/μL) were microinjected into *Drosophila* attP2 embryos (<60 min post-oviposition) using the PhiC31 integrase system. Injected embryos were cultured at 25°C (60–70% humidity, 12:12 light/dark cycle). P0 adults were crossed with w¹¹¹⁸, and F1 miniwhite⁺ individuals backcrossed with TM2/TM6B to stabilize transgenes. For germline-specific expression, UAS-cif/TM6B lines were crossed with Nos-gal4/CyO flies (BDSC 4442), and F2 progeny intercrossed to establish stable lines. The injection and screening of integration flies were conducted by Qidong Fungene Biotechnology (<http://www.fungene.tech>). Transgene integration was verified by PCR. All lines were maintained at 25°C on standard medium.

Mating experiments were conducted with different *Drosophila* lines. The experiments were performed using *Drosophila* culture tubes (containing food). In each tube, two males and one unmated female were placed together for mating. The first observation was made after 2 days of incubation, then all adult flies were removed, and the total number of eggs laid was counted under a stereomicroscope. The culture was then continued for another 2 days, followed by the second observation. The food in the culture tube was filtered through net to separate the larvae, and the number of hatched larvae was recorded to calculate the hatching rate. The crosses were maintained at ∼25 °C on a standard diet. Each combination was repeated in 20 groups.

Supplementary References

Arndt D, et al. 2016. PHASTER: a better, faster version of the PHAST phage search tool. Nucleic Acids Research 44: W16-W21. doi: 10.1093/nar/gkw387

Beckmann JF, Sharma GD, Mendez L, Chen H, Hochstrasser M 2019. The *Wolbachia* cytoplasmic incompatibility enzyme CidB targets nuclear import and protamine-histone exchange factors. eLife 8: e50026. doi: 10.7554/eLife.50026

Bing X-L, et al. 2023. *Wolbachia* manipulates reproduction of spider mites by influencing herbivore salivary proteins. Pest Management Science 79: 315-323. doi: <https://doi.org/10.1002/ps.7201>

Braig HR, Zhou W, Dobson SL, O’Neill SL 1998. Cloning and characterization of a gene encoding the major surface protein of the bacterial endosymbiont *Wolbachia pipientis*. Journal of Bacteriology 180: 2373-2378.

Camacho C, et al. 2009. BLAST+: architecture and applications. BMC Bioinformatics 10: 421. doi: 10.1186/1471-2105-10-421

Cantalapiedra CP, Hernández-Plaza A, Letunic I, Bork P, Huerta-Cepas J 2021. eggNOG-mapper v2: functional annotation, orthology assignments, and domain prediction at the metagenomic scale. Molecular Biology and Evolution 38: 5825-5829. doi: 10.1093/molbev/msab293

Chen L, et al. 2025. Population genomics reveal multiple independent origins of pesticide resistance in the polyphagous pest, *Tetranychus urticae*. Communications Biology 8: 1197. doi: 10.1038/s42003-025-08658-9

Chen S, Zhou Y, Chen Y, Gu J 2018. fastp: an ultra-fast all-in-one FASTQ preprocessor. Bioinformatics 34: i884-i890. doi: 10.1093/bioinformatics/bty560

Cheng H, Concepcion GT, Feng X, Zhang H, Li H 2021. Haplotype-resolved de novo assembly using phased assembly graphs with hifiasm. Nature Methods 18: 170-175. doi: 10.1038/s41592-020-01056-5

De Mendiburu F, Simon R 2015. Agricolae - Ten years of an open source statistical tool for experiments in breeding, agriculture and biology. PeerJ PrePrints 3: e1404v1401. doi: 10.7287/peerj.preprints.1404v1

Gao S, et al. 2024. Transcriptomic landscapes reveal development-related physiological processes in the two-spotted spider mite, *Tetranychus urticae*. Experimental and Applied Acarology 93: 743-759. doi: 10.1007/s10493-024-00956-x

Hong XY, Gotoh T, Nagata T 2002. Vertical transmission of *Wolbachia* in *Tetranychus kanzawai* Kishida and *Panonychus mori* Yokoyama (Acari: Tetranychidae). Heredity 88: 190-196. doi: 10.1038/sj.hdy.6800026

Li H, Durbin R 2009. Fast and accurate short read alignment with Burrows–Wheeler transform. Bioinformatics 25: 1754-1760. doi: 10.1093/bioinformatics/btp324

Li H, et al. 2009. The sequence alignment/map format and SAMtools. Bioinformatics 25: 2078-2079. doi: 10.1093/bioinformatics/btp352

Lindsey ARI, et al. 2018. Evolutionary genetics of cytoplasmic incompatibility genes *cifA* and *cifB* in prophage WO of *Wolbachia*. Genome Biology and Evolution 10: 434-451. doi: 10.1093/gbe/evy012

McKenna A, et al. 2010. The genome analysis toolkit: A MapReduce framework for analyzing next-generation DNA sequencing data. Genome Research 20: 1297-1303. doi: 10.1101/gr.107524.110

Mouton L, Henri H, Bouletreau M, Vavre F 2005. Multiple infections and diversity of cytoplasmic incompatibility in a haplodiploid species. Heredity 94: 187-192. doi: 10.1038/sj.hdy.6800596

Nguyen DT, Morrow JL, Spooner-Hart RN, Riegler M 2017. Independent cytoplasmic incompatibility induced by *Cardinium* and *Wolbachia* maintains endosymbiont coinfections in haplodiploid thrips populations. Evolution 71: 995-1008. doi: 10.1111/evo.13197

Patro R, Duggal G, Love MI, Irizarry RA, Kingsford C 2017. Salmon provides fast and bias-aware quantification of transcript expression. Nature Methods 14: 417. doi: 10.1038/nmeth.4197

R: a language and environment for statistical computing [Internet]. Vienna, Austria: R Foundation for Statistical Computing; 2024. Available from: <http://www.R-project.org/>

Shen W, Le S, Li Y, Hu F 2016. SeqKit: a cross-platform and ultrafast toolkit for FASTA/Q file manipulation. PLoS ONE 11: e0163962. doi: 10.1371/journal.pone.0163962

Siguier P, Perochon J, Lestrade L, Mahillon J, Chandler M 2006. ISfinder: the reference centre for bacterial insertion sequences. Nucleic Acids Research 34: D32-D36. doi: 10.1093/nar/gkj014

Sun J-X, et al. 2016. Effects of host interaction with *Wolbachia* on cytoplasmic incompatibility in the two-spotted spider mite *Tetranychus urticae*. Biological Journal of the Linnean Society 119: 145-157. doi: 10.1111/bij.12804

Tatusova T, et al. 2016. NCBI prokaryotic genome annotation pipeline. Nucleic Acids Research 44: 6614-6624. doi: 10.1093/nar/gkw569

gggenes: draw gene arrow maps in 'ggplot2' [Internet]. 2023. Available from: <https://wilkox.org/gggenes/>

Xie R-R, Chen X-L, Hong X-Y 2011. Variable fitness and reproductive effects of *Wolbachia* infection in populations of the two-spotted spider mite *Tetranychus urticae* Koch in China. Applied Entomology and Zoology 46: 95-102. doi: 10.1007/s13355-010-0014-x

Zimmermann L, et al. 2018. A completely reimplemented MPI bioinformatics toolkit with a new HHpred server at its core. Journal of Molecular Biology 430: 2237-2243. doi: <https://doi.org/10.1016/j.jmb.2017.12.007>

Supplementary Figures

Fig. S1. Experimental design for unifying nuclear genetic background through multiple generations of backcrossing.

Experimental design for multiple generations of backcrossing to unify the nuclear genetic background of *Tetranychus urticae*. The following steps were implemented: Initially, female WI from the JN population (green, *Wolbachia*-infected) were crossed with male WU from the NM population (white, *Wolbachia*-free) to generate F1 offspring. From the F1 generation to F8, each subsequent generation was backcrossed using new generated NJ WI females and NM WU males, gradually replacing the nuclear genes with the NM nuclear background. *Wolbachia* (indicated by circles) was maternally inherited and stably transmitted through all generations. After eight generations of backcrossing, the nuclear genetic background of the NJ population was replaced with the NM background, while *Wolbachia* from the JN population was retained.

Fig. S2. Representative predicted structures of CifA (A) and CifB (B) proteins.

Representative structures are shown for each type, with the *Wolbachia* strain name, accession number, and length of the protein indicated at the N-terminus. The arrows on cifB indicates where we cloned for functional experiments in yeast and *Drosophila* studies.

Fig. S3. Multiple sequence alignment (MSA) of the CifB PD-(D/E)XK domain.

The CifB sequences in MSA are labelled by their types (2, …, 8) and the REase domain (4) followed by their species abbreviation and NCBI accession number. The typical catalytic residues of the REase are highlighted in red font is shown above the MSA.

The type I CifB protein that lack the characteristic active site residues is listed at the bottom.

Fig. S4. Sequence alignment depth of the *cif* gene operon regions in *w*Turt genome.

(A) Sequence alignment of the *cifA1* - *cifB1* operon region in *w*Turt. The top of the panel shows the target region's position and coverage in the *w*Turt genome CP114181, spanning from 1,201,531 to 1,209,857 bp (covering 8,328 bp). (B) Sequence alignment of the *cifA2* - *cifB2* operon region in *w*Turt. The top of the panel shows the target region's position and coverage in the *w*Turt genome CP114181, spanning from 1,307,822 to 1,316,864 bp (covering 9,044 bp). In each subplot, multiple gray tracks represent different samples (with population names indicated next to the tracks). The “peaks and valleys” within each track reflect the sequencing depth and read alignment support at corresponding genomic positions.

Fig. S5. Expression patterns and correlation analysis of *wsp* and *cif*-related genes of *w*Turt across different *T. urticae* populations.

(A) Expression levels of the *Wolbachia* *wsp* gene in female spider mites from different populations, normalized as *wsp/rps18*. (B) Correlation analysis of *Wolbachia* density (indicated by *wsp/rps18*) in female and male mites from different populations. (C) Expression levels of each *cif* gene in female *T. urticae* infected with *w*Turt, normalized as *cif/wsp*. Different letters indicate significant statistical differences of One-way ANOVA analysis (p < 0.05). (D) Correlation analysis between the expression of *cifA* and *cifB* in females (*cif/wsp*). Pair1 (*cifA1/cifB1*) is represented by red circles, and Pair2 (*cifA2/cifB2*) is represented by blue triangles. (E) Correlation analysis of *cifB2* expression and *Wolbachia* density (indicated by *wsp/rps18*) in males and females. Males are represented by blue triangles, and females by red circles. The regression equations and *p* values for correlation analyses are displayed in the figure.

Fig. S6. Toxicity growth assay in yeast expressing *w*Turt *cifA* and *cifB* alleles.

(A) Yeast cells were transformed with pRS425GAL1 and pYES2 plasmids, which express *cifA* and *cifB*, respectively. EV represents empty vector. Yeast cells were serially diluted (5-fold) and spotted on selective plates with specific carbon sources, followed by incubation at 30°C and 36°C for 2.5 days. The # next to cifB indicates that CifB consists solely of the PDDEXK domain (B).

Fig. S7. RT-PCR analysis of *wTurt* cif gene expression in transgenic *Drosophila*.

Gel electrophoresis showing the PCR products of *w*Turt *cif* gene expression in transgenic *D. melanogaster*.

# Supplementary Tables

Table S1. Spider mite sample information

Table S2. Primers used in this study

Table S3. WO regions predicted by PHASTER in *w*Turt

Table S4. Filtered SNP effects for *Wolbachia* from different mite populations
